# Enhanced Storage Stability and Rheological Properties of Asphalt Modified by Activated Waste Rubber Powder

**DOI:** 10.3390/ma14102693

**Published:** 2021-05-20

**Authors:** Weihong Liu, Yishen Xu, Hongjun Wang, Benan Shu, Diego Maria Barbieri, Jose Norambuena-Contreras

**Affiliations:** 1 Jiangsu College of Engineering and Technology, Nantong 226007, China; lwh-81@163.com (W.L.); hjwang_nt@163.com (H.W.); 2 Foshan Transportation Science and Technology Co., Ltd., Foshan 528000, China; xuyishen0603@126.com; 3 Department of Civil and Environmental Engineering, Norwegian University of Science and Technology, 7491 Trondheim, Norway; diego.barbieri@ntnu.no; 4 LabMAT, Department of Civil and Environmental Engineering, Universidad del Bío-Bío, Concepcion 1202, Chile; jnorambuena@ubiobio.cl

**Keywords:** waste rubber powder, microwave activation, chemical grafting, modified asphalt, storage stability, rheological properties

## Abstract

Segregation of waste crumb rubber powder (WR) modified asphalt binders the large-scale application of WR in asphalt. The method of microwave activation combined with chemical activation (KMWR) was proposed to improve storage stability and rheological properties of WR modified asphalt in this work. Storage stability and rheological properties of virgin asphalt, MWR modified asphalt, and KMWR modified asphalt were comparatively studied by the standard segregation test, bending beam rheometer (BBR) test, and dynamic shear rheometer (DSR) test. The effect of composite activation on waste rubber powder particles was studied by Fourier-transform infrared spectroscopy (FT-IR), scanning electron microscope (SEM), and Brunauer–Emmett–Teller (BET) tests. The main results showed that after the physical and chemical composite activation, the storage stability of waste rubber powder modified asphalt was significantly improved, WR modified asphalt had better crack resistance, better rutting resistance, and better fatigue performance. After physical and chemical activation, WR was desulfurized, and a large number of active groups was grafted on the WR particles.

## 1. Introduction

As a kind of high molecular polymer, rubber is widely used in tire products. With the failure of rubber tires, these solid wastes put a lot of pressure on the environment [1,2]. Waste rubber tires have been used to produce more sustainable construction materials with thermophysical and energy harvesting purposes [3,4,5]. Asphalt pavement has been built on a large scale due to the comfortable driving experience [6,7]. In particular, the waste rubber tires can be recycled and crushed into crumb to produce rubber powder modified asphalt for environment-friendly construction [8]. Several works have been done on waste crumb rubber modified asphalt (WRMA) [9,10,11,12,13,14]. It was proved that the addition of waste crumb rubber (WR) could improve the performance of asphalt, such as better rutting resistance [15,16,17], enhanced fatigue life [18,19], and moisture damage resistance [20].

However, the rubber existing in waste tires is highly cross-linked by disulfide bonds. The movement of rubber molecules is thus limited, which makes the rubber surface inert. This cross-linked structure makes the compatibility between rubber and asphalt worse [21,22]. In addition, more than 20 wt.% carbon black is added into tires to improve tire strength, so a large number of inorganic materials such as carbon black were fixed in rubber molecules, and the compatibility between rubber powder and asphalt was further weakened [23]. Therefore, compatibility and segregation have been plaguing the large-scale application of WRMA. Many research have been done to solve this problem [24]. So far, three kinds of methods have been tried to solve this problem [25,26,27].

The first method is the physical method is about desulfurization of WR particles by external stimulation, which includes increased stirring rates and mixing temperatures [28]. Li et al. [29] found that mixing time was more effective than mixing temperature for improving the storage stability of WRMA, but for desulfurization, mixing temperature played a decisive role. Billiter et al. [30] revealed that the low temperature performance of WRMA obtained at 260 °C was greatly improved, but the high temperature performance was weakened.

The second method is the chemical method. Trans-polyoctenamer (TOR) has been used to connect WR with asphalt [31,32]. It revealed that the addition of TOR improved the compatibility of WR in asphalt by the cross-linking action of sulfides between WR and asphaltenes in asphalt. Kocevski et al. [33] pointed out that the bulk polymerization of acrylic acid with rubber particles improved viscosity of rubber powder modified asphalt. Xie et al. [34] found that surface grafting modification of acrylamide on rubber particles improved compatibility and storage stability of rubber powder modified asphalt.

In recent years, as one of the three methods, the activation method has attracted a lot of attention because of its good effect and low energy consumption. Kang et al. [35] found that microwave activation successfully broke the S–S bond in rubber particles. Conventional properties and storage stability of rubber modified asphalt were improved. Liang et al. [36] studied the effect of microwave activated rubber powder on fume emission and rheological properties of asphalt. Results indicated that the addition of microwave activated rubber powder decreased the emission of sulfides and PAHs. In addition, compared with unactivated rubber modified asphalt, activated rubber powder decreased rutting resistance of asphalt at high temperature.

To make WR desulfurized and stronger activated, this work proposed a method about microwave activation combined with chemical grafting on WR. The WR was firstly activated by microwaves action, the sample was named as MWR. Due to the non-polarity and existence of massive inorganic substances (like carbon black), the MWR was then grafted by the silane coupling agent KH550, and the sample was named as KMWR. Segregation, Brookfield viscosity, and dynamic shear rheometer (DSR) tests were conducted to investigate storage ability and rheological properties of WRMA. Effect of MCA on WR was studied by Fourier-transform infrared spectroscopy (FT-IR), scanning electron microscope (SEM), and Brunauer–Emmett–Teller (BET) tests.

## 2. Materials and Methods

### 2.1. Materials

**Figure 1:**
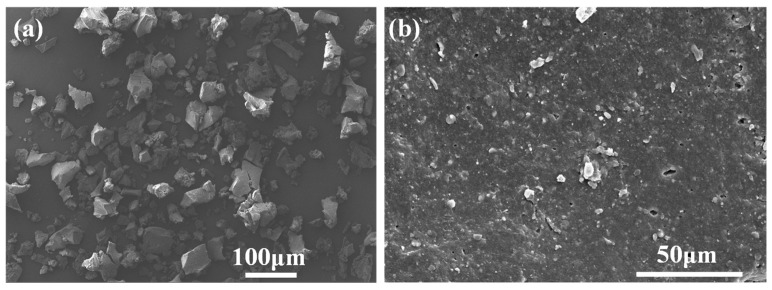
SEM images of waste tire crumb rubber particles under different magnifications: (**a**) ×50; (**b**) ×1000.

WR was bought from Dujiangyan Huayi Rubber Co. Ltd. (Wuhan, China). The micromorphology of the WR was tested and the SEM image is shown in Figure 1. The diameter of WR is about 106 μm (150 mesh). The 70# asphalt was supplied by KOCH Asphalt Co. Ltd. (Wuhan, China), and its penetration (20 °C, 0.1 mm), softening point and ductility (15 °C) were 68.5, 48.2 °C, and 148 cm, respectively. The test was carried out according to the specification JTG E20-2011 (standard test methods of bitumen and bituminous mixtures for highway engineering, Beijing, China). The silane coupling agent KH550 was provided by Sinopharm Chemical Reagent Co. Ltd. (Wuhan, China)

### 2.2. Experimental Methods

The WR was firstly activated by a microwave oven, the microwave frequency and power were 2450 MHz and 700 W, respectively. Then, the MWR was grafted by KH550. The morphology of the three kinds of WR was observed by SEM (QUANTA FEG 450 (FEI, WA, USA). The chemical structure of the three kinds of WR was tested by FTIR (Thermo Nicolet, WA, USA), the tested wavelength was from 400 to 4000 cm^−1^.

The three kinds of asphalt specimens, which included the asphalt containing 15 wt.% WR, the asphalt containing 15 wt.% MWR, and the asphalt containing 15 wt.% KMWR were prepared. The preparation process was the same as that in former work [37]. The mixing temperature, mixing speed and mixing time was controlled as 160 °C, 5000 rpm, and 40 min respectively.

A segregation test was performed at 160 °C for 48 h. The softening point test was conducted on the upper 1/3 part asphalt (S_U_) and the bottom 1/3 part asphalt (S_B_). The softening point difference (S_D_) was used to evaluate storage stability of WRMA. The segregation test was conducted according to the specification JTG E20-2011, China (standard test methods of bitumen and bituminous mixtures for highway engineering).

The Brookfield viscosity (Brookfield, WA, USA) test was performed to explore the viscosity change of asphalt with temperature. Rotor of 27# was used. The viscosity at four temperatures was tested and recorded, which includes 80 °C, 95 °C, 110 °C, and 125 °C. The temperature was raised every half an hour. The test was carried out according to the specification JTG E20-2011, China (standard test methods of bitumen and bituminous mixtures for highway engineering).

The BBR (Cannon, Oita Prefecture, Japan) test was used to evaluate crack resistance of WRMB at low temperature. When the test time reached 60 s, the stiffness and m values were recorded at this time. Specific operation steps were carried out in accordance with AASHTO T 313 specification.

The DSR (Anton Paar, Canberra, Australia) test was conducted to study high-temperature rutting resistance of WRMA. The temperature sweep with a constant frequency of 10 rad/s was performed under the strain-controlled mode. The performance parameters complex shear modulus ($G^{*}$) and phase angle (δ) could be obtained. The rutting resistance of WRMA was evaluated by rutting factor $G^{*}/\sin\delta$. Specific operation steps were carried out in accordance with AASHTO T 315 specification.

Multiple stress creep recovery (MSCR) test was performed according to the AASHTO T 350 specification. Shear stress and test temperature were controlled at 300 Pa and 40 °C respectively. Loading period and rest period were 1 s and 5 s, respectively.

According to the reference [38], fatigue test with the time sweep mode was performed to study the effect of MCA on antifatigue damage of WRMA. The loading strain and rate were 3% and 10 Hz, respectively. The test temperature was kept at 15 °C. When the damage degree reached 50% (namely the modulus was reduced to half of the initial modulus, 0.5$G_{i}$), the fatigue cycle, namely fatigue life $N_{i}$ was obtained. Three sets of replicate specimens were tested for rheological tests.

## 3. Results and Discussion

### 3.1. Segregation Result of Waste Rubber Powder Modified Asphalt

The storage stability of different kinds of waste rubber powder modified asphalt is shown in Table 1. The smaller the difference in softening point, the better the storage stability of waste rubber powder modified asphalt, and the better compatibility of waste rubber powder in asphalt.

It can be seen from Table 1 that the storage stability of WR modified asphalt was the worst, followed by MWR modified asphalt. The difference in softening point of KMWR modified asphalt was 1.8 °C. The results indicated that on the base of microwave activation, MCA could further enhance the storage stability of waste rubber powder modified asphalt. The result may be attributed that microwave activation made the WR particles desulfurized. The desulfurized WR particles had a better swell property in asphalt, so the storage stability of WR modified asphalt was improved. After MCA, a large number of active groups could be grafted on the microwave activated WR. Strong intermolecular forces could be formed between WR particles and asphalt molecules. WR particles contain more than 20 wt.% carbon black, so strong intermolecular forces also can be formed between inorganic materials (carbon black) and asphalt. Therefore, the storage stability of KMWR modified asphalt was the best.

### 3.2. Rheological Properties of Waste Rubber Powder Modified Asphalt

#### 3.2.1. Bending Beam Rheometer (BBR) Test

**Figure 2:**
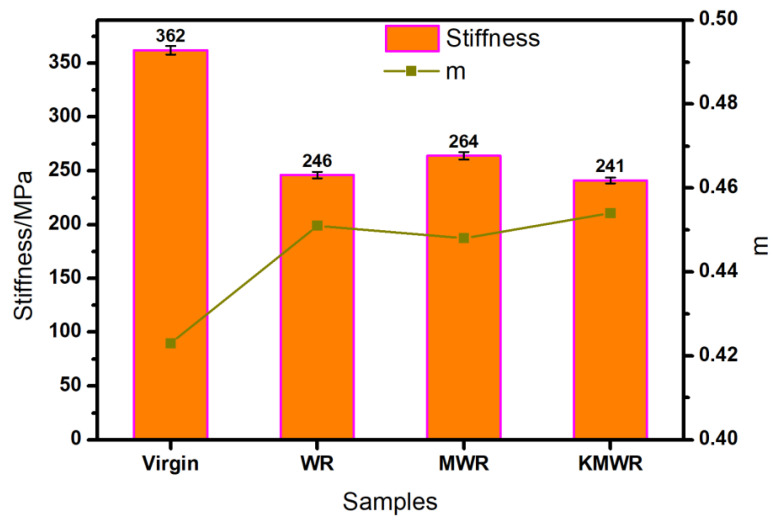
Stiffness and “m” values of the four kinds of asphalt.

The bending beam rheometer (BBR) test was performed to evaluate crack resistance of WRMA at low temperature, and the result is shown in Figure 2. The stiffness of virgin asphalt was larger than 300 MPa at −16 °C, so −16 °C was determined as the critical temperature. Stiffness describes the brittleness degree of asphalt. The smaller stiffness, the better crack resistance of asphalt.

It can be found from Figure 2 that the addition of waste rubber powder significantly decreased the stiffness of virgin asphalt. The stiffness and “m” values of WRMA were 246 MPa and 0.451 respectively, which means that waste rubber powder improved crack resistance of virgin asphalt at low temperature. The stiffness value had a slight decrease and the “m” had a slight increase compared with MWR modified asphalt, which indicated that the crack resistance of KMWR modified asphalt was better than that of MWR modified asphalt.

#### 3.2.2. Brookfield Viscosity Test

**Figure 3:**
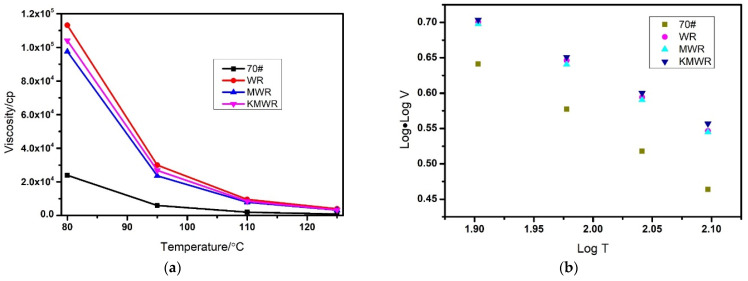
Effect of temperature on viscosity-temperature curves (**a**) and Log relation (**b**) of the four kinds of asphalt.

The viscosity–temperature characteristic of the four kinds of asphalt is shown in Figure 3. From Figure 3a, it can be found that the viscosity of all kinds of asphalt specimens decreased with the increase of temperature, which was attributed to the intrinsic viscoelasticity. In addition, the viscosity of WRMA was over five times larger than that of virgin asphalt, which indicated that the addition of waste rubber particles significantly improved rutting resistance of asphalt at high temperature. Compared with MWR, the viscosity of KMWR modified asphalt had a little increase.

The logarithmic relation of viscosity–temperature of asphalt is shown in Figure 3b, the slope fitting value is viscosity temperature susceptibility (VTS), and the result is shown in Table 2. VTS reflects temperature susceptibility of asphalt. The smaller absolute value of VTS, the smaller the variation in the performance of asphalt on temperature. The order of VTS was: KMWR < MWR < WR < 70#, which revealed that the addition of waste rubber powder reduced the temperature sensitivity of asphalt. Compared with MWR, KMWR could further decrease the temperature sensitivity of asphalt.

#### 3.2.3. Temperature Sweep Test

**Figure 4:**
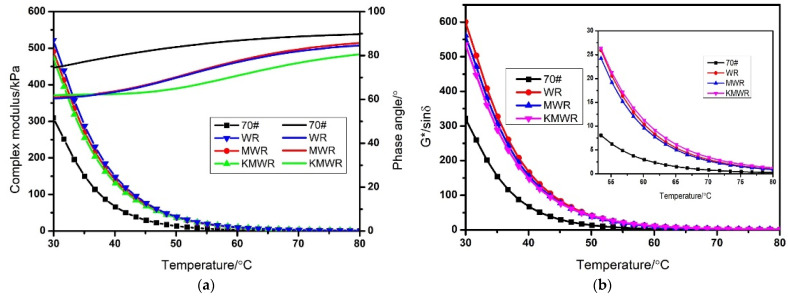

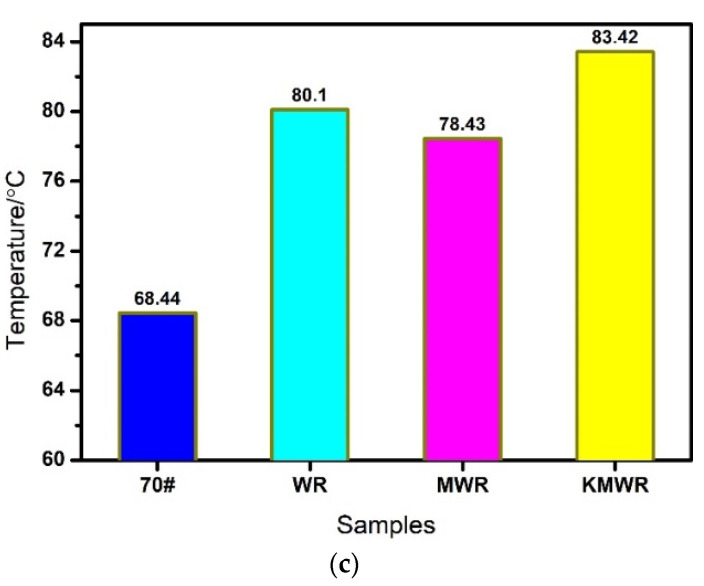
Effect of temperature on complex modulus and phase angles (**a**), rutting factor, (**b**) and failure temperature (**c**) of the four kinds of asphalt.

The change of the complex modulus and phase angle of asphalt with temperature was studied by the DSR test, and the result is presented in Figure 4a. It demonstrated that the three kinds of waste rubber powder improved the complex modulus of asphalt. The phase angle of three kinds of modified asphalt showed a decrease compared with virgin asphalt. The rutting factor reflects the rutting resistance of asphalt pavement at high temperature. It can be found from Figure 4b that when temperature was less than 55 °C, the order of rutting factors was WR > MR > KMWR > 70#. When the temperature was over 55 °C, the order of rutting factors was KMWR > WR > MWR > 70#. The result revealed that the composite activation on waste rubber powder made asphalt had the best rutting resistance (when the temperature is over 55 °C).

The temperature at which the rutting factor is less than 1 kPa is called the failure temperature, which is used to evaluate the performance grade of asphalt [39]. From Figure 4c, it can be seen that the addition of waste rubber powder significantly improved the failure temperature of virgin asphalt. It is noted that the composite activation further improved failure temperature of asphalt compared with MWR modified asphalt.

#### 3.2.4. Multiple Stress Creep Recovery (MSCR) Test

**Figure 5:**
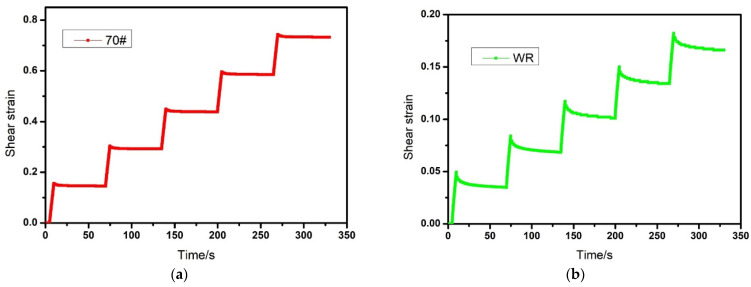

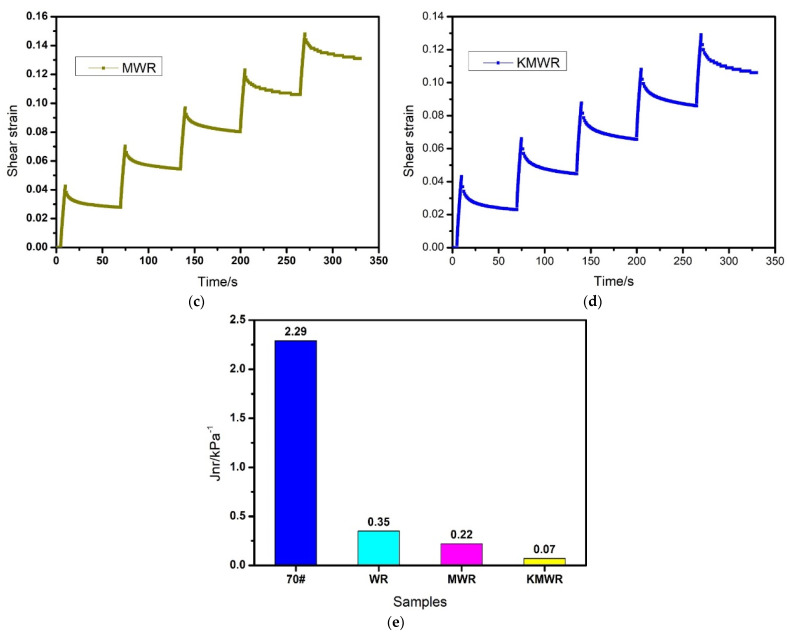
Effect of repeated loading-unloading cycles on shear strain and non-recoverable creep compliance (**e**) of virgin asphalt (**a**), WR modified asphalt (**b**), MWR modified asphalt, (**c**) and KMWR modified asphalt (**d**).

In order to realistically simulate the antideformation performance of asphalt pavement under the traffic loading, the MSCR test was conducted, and the result is shown in Figure 5. From Figure 5a–d, it can be seen that after five loading cycles, the cumulative deformation of virgin asphalt, WR, MWR, and KMWR modified asphalt were 0.732, 0.166, 0.131, and 0.106 respectively. It meant that the composite activation could significantly decrease the deformation of WRMA when suffering repeated shear stress. Non-recoverable creep compliance $J_{nr}$ was calculated to quantitatively evaluate deformation resistance of asphalt:

(1) $$J_{nr}=\frac{\gamma_{nr}}{\tau }$$

where, τ means shear stress; $\gamma_{nr}$ means residual strain after *n* numbers of loading–unloading cycles.

From Figure 5e, it could be found that $J_{nr}$ was significantly decreased after the addition of waste rubber powder. The $J_{nr}$ of KMWR modified asphalt was the smallest, followed by MWR and WR modified asphalt. The result indicated that the elastic characteristic of WR made asphalt had an excellent deformation recovery ability. Compared with MWR, KMWR further improved elastic recovery of asphalt. It may be attributed that after composite activation, strong intermolecular forces could be formed between waste rubber powder and asphalt. These forces just like “bridges”, which tightly connect the waste rubber powder (including massive inorganic carbon black) and asphalt. Therefore, KMWR modified asphalt had the best elastic recovery ability. Microwave activation did not create such a “bridge” between WR and asphalt, thus the elastic recovery ability of KMWR modified asphalt was better than that of MWR modified asphalt.

#### 3.2.5. Fatigue Test

**Figure 6:**
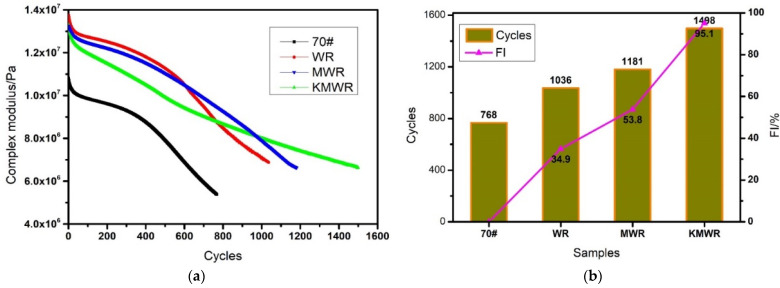
Effect of loading cycles on complex modulus (**a**) and fatigue life (**b**) of the four kinds of asphalt.

Fatigue failure will generate inside asphalt pavement under the traffic loading. After the generation of fatigue microcracks, the performance of asphalt pavement will be deteriorated rapidly under the action of external factors, such as rainwater and oxygen. In this study, the fatigue test was conducted on a DSR machine to evaluate the effect of waste rubber powder on fatigue performance of asphalt, and the result is shown in Figure 6.

From Figure 6a, it was revealed that the addition of the three kinds of WR significantly increased the initial complex modulus of asphalt. With the increase of fatigue loading cycles, the complex modulus of the four kinds of asphalt showed different decreasing trend. In the initial loading stage, the modulus of WR modified asphalt was the largest, followed by the MWR modified asphalt and KMWR modified asphalt. With the increase of loading cycles, the modulus of WR modified asphalt had the fastest decrease, and the modulus of KMWR modified asphalt had the least decrease. The result indicated that the “bridge” effect between WR and asphalt was not strong, so that under the effect of fatigue loading, the interface between WR and asphalt was most prone to generate cracks, which induces fatigue failure. Microwave activation made WR particles desulfurized and de-crosslinked, the compatibility of WR in asphalt was thus improved, so the modulus of MWR modified asphalt had a less reduction than that of WR modified asphalt under the action of fatigue loading. Synergistic “desulfurization” and “bridge” effect significantly improved fatigue damage resistance of KMWR modified asphalt. From Figure 6b, it can be seen that the addition of WR significantly increased the fatigue life of asphalt. Compared with virgin asphalt, the fatigue life of WR, MWR and KMWR modified asphalt was increased by 34.9%, 53.8%, and 95.1% respectively. It revealed that the “desulfurization” and “bridge” worked and improved the fatigue performance of waste rubber powder modified asphalt.

### 3.3. Effect of Composite Activation on Waste Rubber Powder

#### 3.3.1. FT-IR Test

**Figure 7:**
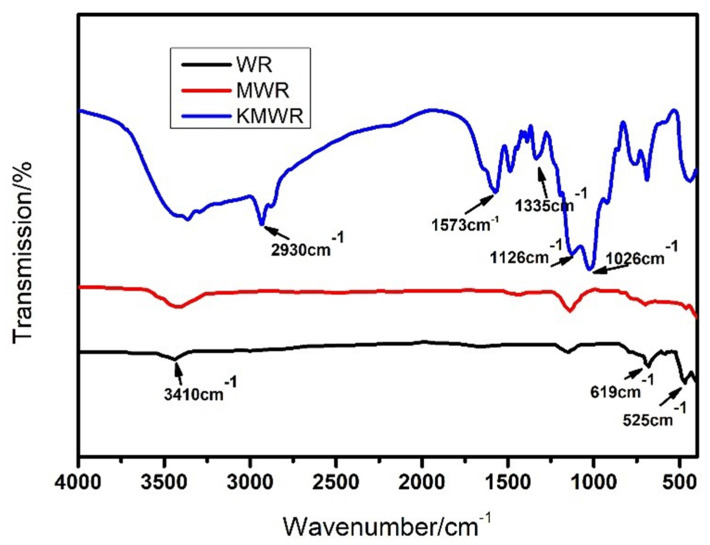
FTIR results of the three kinds of waste rubber powder.

The FT-IR test was used to study the difference in chemical bonds and functional groups of the three kinds of WR. The characteristic peak intensity of S–S and C–S reflect the crosslinking degree of WR. As shown in Figure 7, the characteristic peak at 525 cm^−1^ is due to the vibration of S=S bond, and the characteristic peak at 619 cm^−1^ is due to the vibration of C–S bond. It can be seen that after microwave activation, the characteristic peak intensity of S=S and C-S significantly decreased, which means that microwave activation had a desulfurization effect on waste rubber powder, and the cross-linking degree of WR was decreased. After the microwave activation, the intensity of characteristic peak –OH at 3410 cm^−1^ was slightly increased. After composite activation, more active groups were formed on MWR. 1026 cm^−1^ and 1126 cm^−1^ were peaks associated with vibration of C-N, which reveals that microwave activated WR were successfully grafted by KH550.

In addition, the peak strength of oxygen groups was further increased. It indicated that reactivity of KMWR was significantly enhanced. 1573 cm^−1^ was symmetrical stretching of N–H, and 1335 cm^−1^ was in-plane bending of N-H. The two kinds of active hydrogens on amino groups act as “bridge” to connect the non-polar substances (such as WR and carbo black) and asphalt. Based on the FT-IR result, it can be concluded that the composite activation destroyed the vulcanization structure, as well improved the reactivity of waste rubber powder. In particular, the mass ratio of carbon black to rubber was close to 1:2 in waste rubber tire crushed particles [40]. Strong intermolecular forces could be formed between carbon black and asphalt after the composite activation, thus the storage stability of waste rubber powder modified asphalt can be further improved.

#### 3.3.2. SEM Test

**Figure 8:**
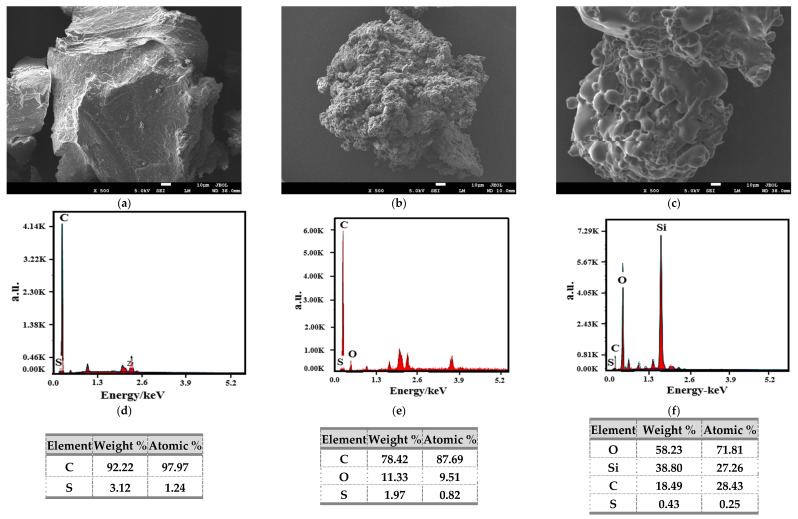
SEM images (**a**–**c**) and element distribution (**d**–**f**) of the three kinds of waste rubber powder.

The effect of the composite activation on micromorphology of waste rubber powder was studied, and the result is shown in Figure 8. Figure 8a revealed that the surface of WR was smooth and dense. Figure 8d showed that the main elements of WR were carbon and sulfur. After the microwave activation, from Figure 8b it can be found that the surface of MWR was rougher, a large number of bulges were formed, and the WR particles became fluffy. It indicated that the internal structure of WR was changed after the action of microwave activation.

Figure 8e showed that the main elements of MWR were carbon, oxygen, and sulfur. The result revealed that microwave activation made WR desulfurized, and a small amount of active groups were formed on the MWR. The activity could improve the storage stability of WRMA, but the activation ability of MWR was still weak (based on the segregation result). After the composite activation, the waste rubber powder was covered with a thin film.

From Figure 8f, it illustrated that the main elements of KMWR were oxygen, silicon, carbon, and sulfur. The result means that the film was silane coupling agent KH550. The coupling agent was successfully grafted onto KMWR, and a large amount of active groups were grafted on the surface. So, the storage stability of KMWR modified asphalt was further improved.

#### 3.3.3. BET Test

**Figure 9:**
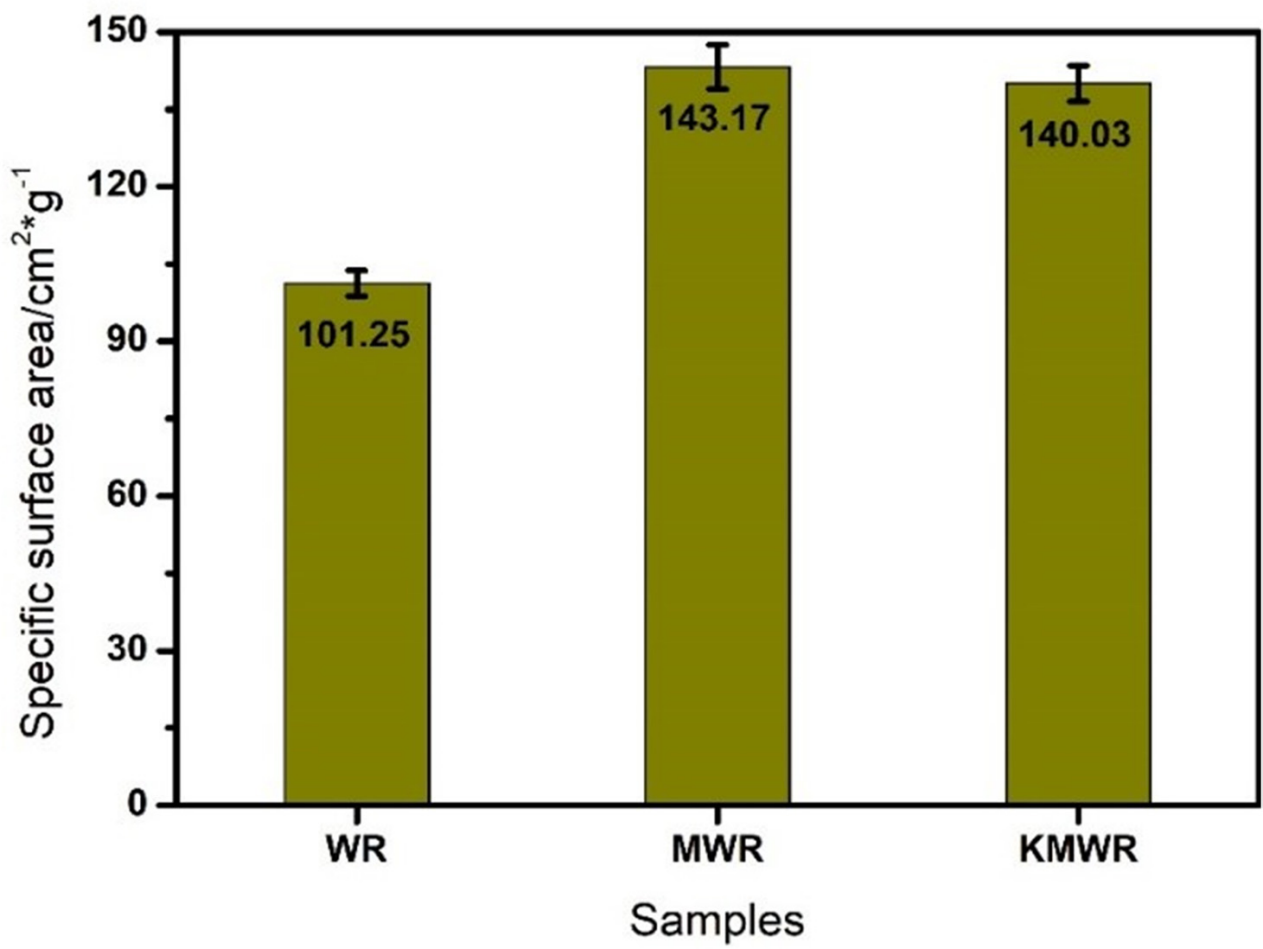
Specific surface area of the three kinds of waste rubber powder.

The effect of activation on specific surface area of WR is shown in Figure 9. It can be seen that, after composite activation, the specific surface area of WR was increased from 101.25 to 140.03 cm^2^/g. The result revealed that MCA destroyed the cross-linking structure of WR and enhanced the specific surface area of WR particles.

Based on the above analysis, it can be found that the composite activation had a desulfurization effect on waste rubber powder. The increased specific surface area made the waste rubber powder have the better swelling effect in asphalt. In addition, massive active groups were grafted on waste rubber powder, strong intermolecular forces can be formed between waste rubber powder and asphalt, which act like a “bridge”, tightly connected WR and asphalt.

## 4. Conclusions

This work proposed the physical and chemical composite activation on waste rubber powder. Storage stability and rheological properties of three kinds of modified asphalt were comparatively studied. In addition, the effect of composite activation on morphology and microstructure of WR was investigated. Based on the above results and analysis, the following conclusions can be drawn:

(1) The physical and chemical composite activation on waste rubber powder particles significantly improved the storage ability of WR modified asphalt, the softening point difference of KMWR modified asphalt was 1.8 °C.
(2) KMWR modified asphalt had better crack resistance, better elastic recovery, better fatigue damage resistance, and higher failure temperature. After the physical and chemical composite activation, the stiffness of WR modified asphalt was decreased from 362 to 241 MPa, the non-recoverable creep compliance of WR modified asphalt was decreased from 2.29 to 0.07 kPa^−1^, The fatigue life of WR modified asphalt was nearly doubled than that of virgin asphalt.
(3) The composite activation made waste rubber powder desulfurized and grafted a large number of active groups, such as –OH and –N-H.

## Figures and Tables

**Table 1:** Segregation result of the three kind of waste rubber powder modified asphalt.

Asphalt Types	S_B_	S_U_	S_D_
WR	67.6	61.5	6.1
MWR	67.2	63.7	3.5
KMWR	66.7	64.9	1.8

**Table 2:** Viscosity temperature susceptibility of the four kinds of asphalt.

Samples	70#	WR	MWR	KMWR
VTS	−0.9155	−0.7951	−0.7899	−0.7606
R^2^	0.9992	0.9984	0.9997	0.9992

## Data Availability

Data sharing not applicable.

## Abbreviations

WR	waste crumb rubber powder
MCA	Microwave and chemical composite activation
KMWR	The WR which was firstly activated by microwave, and then was grafted by the silane coupling agent KH550
MWR	The WR that was activated by a microwave
WRMA	WR modified asphalt
